# Computer-controlled closed-loop norepinephrine infusion system for automated control of mean arterial pressure in dogs under isoflurane-induced hypotension: a feasibility study

**DOI:** 10.3389/fvets.2024.1374356

**Published:** 2024-05-31

**Authors:** Kazumasu Sasaki, Toru Kawada, Hiroki Matsushita, Shohei Yokota, Midori Kakuuchi, Aimi Yokoi, Yuki Yoshida, Hidetaka Morita, Kei Sato, Takuya Nishikawa, Annette P. N. Kutter, Yasuyuki Kataoka, Joe Alexander, Keita Saku, Tatsuya Ishikawa, Kazunori Uemura

**Affiliations:** ^1^Akita Cerebrospinal and Cardiovascular Center, Research Institute for Brain and Blood Vessels, Akita, Japan; ^2^Department of Cardiovascular Dynamics, National Cerebral and Cardiovascular Center, Suita, Japan; ^3^Sendai Animal Care and Research Center, Sendai, Japan; ^4^Department of Research Promotion and Management, National Cerebral and Cardiovascular Center, Suita, Japan; ^5^Section of Anesthesiology, Department of Clinical Diagnostics and Services, Vetsuisse Faculty, University of Zurich, Zurich, Switzerland; ^6^Medical and Health Informatics, NTT Research, Inc., Sunnyvale, CA, United States; ^7^NTTR-NCVC Bio Digital Twin Center, National Cerebral and Cardiovascular Center, Suita, Japan

**Keywords:** arterial pressure control, automated control, closed-loop drug infusion system, dog, isoflurane-induced hypotension, norepinephrine

## Abstract

**Introduction:**

Intra-operative hypotension is a common complication of surgery under general anesthesia in dogs and humans. Computer-controlled closed-loop infusion systems of norepinephrine (NE) have been developed and clinically applied for automated optimization of arterial pressure (AP) and prevention of intra-operative hypotension in humans. This study aimed to develop a simple computer-controlled closed-loop infusion system of NE for the automated control of the mean arterial pressure (MAP) in dogs with isoflurane-induced hypotension and to validate the control of MAP by the developed system.

**Methods:**

NE was administered via the cephalic vein, whereas MAP was measured invasively by placing a catheter in the dorsal pedal artery. The proportional-integral-derivative (PID) controller in the negative feedback loop of the developed system titrated the infusion rate of NE to maintain the MAP at the target value of 60 mmHg. The titration was updated every 2 s. The performance of the developed system was evaluated in six laboratory Beagle dogs under general anesthesia with isoflurane.

**Results:**

In the six dogs, when the concentration [median (interquartile range)] of inhaled isoflurane was increased from 1.5 (1.5–1.5)% to 4 (4–4)% without activating the system, the MAP was lowered from 95 (91–99) to 41 (37–42) mmHg. In contrast, when the concentration was increased from 1.5 (1.0–1.5)% to 4 (4–4.8)% for a 30-min period and the system was simultaneously activated, the MAP was temporarily lowered from 92 (89–95) to 47 (43–49) mmHg but recovered to 58 (57–58) mmHg owing to the system-controlled infusion of NE. If the acceptable target range for MAP was defined as target MAP ±5 mmHg (55 ≤ MAP ≤65 mmHg), the percentage of time wherein the MAP was maintained within the acceptable range was 96 (89–100)% in the six dogs during the second half of the 30-min period (from 15 to 30 min after system activation). The median performance error, median absolute performance error, wobble, and divergence were − 2.9 (−4.7 to 1.9)%, 2.9 (2.0–4.7)%, 1.3 (0.8–1.8)%, and − 0.24 (−0.34 to −0.11)%·min^−1^, respectively. No adverse events were observed during the study period, and all dogs were extubated uneventfully.

**Conclusion:**

This system was able to titrate the NE infusion rates in an accurate and stable manner to maintain the MAP within the predetermined target range in dogs with isoflurane-induced hypotension. This system can be a potential tool in daily clinical practice for the care of companion dogs.

## 1 Introduction

Intra-operative hypotension is a common complication observed in dogs undergoing surgery under general anesthesia (1, 2). Intra-operative hypotension has been known to increase the incidence of postoperative complications in humans (3–7). Thus, the incidence of hypotension should be avoided—also in dogs—to enhance the recovery after general anesthesia (8).

Infusion of vasopressors, along with fluid therapy and titration of anesthetic agents, has been performed to prevent the incidence of intra-operative hypotension and maintain an optimally targeted arterial pressure (AP) during surgery. However, manual adjustment of the infusion rates of the vasopressors to achieve the targeted AP is difficult in some cases (9). Consequently, several computer-controlled closed-loop vasopressor infusion systems have been developed for the accurate maintenance of AP in humans (10, 11). The use of these systems in humans has facilitated the maintenance of AP within a predetermined target range with an accuracy equivalent to or better than that of manual titration (12, 13). These results suggest that closed-loop control of the infusion of vasopressors may also be effective in dogs.

In our previous study, a closed-loop automated infusion system of norepinephrine (NE) and fluid was developed for the simultaneous control of MAP and cardiac output in dogs with experimentally induced endotoxin shock. That system was effective in rescuing hemodynamics (14, 15). However, that system was primarily developed for the management of patients with septic shock. Thus, the experimental preparations and protocols in previous studies simulated those used in intensive care settings. Moreover, monitoring of cardiac output and central venous pressure, in addition to MAP, was required, and drugs were infused via the femoral vein, i.e., a central vein. These practices are not suitable for the treatment of companion dogs in veterinary clinics, wherein the cardiac output and central venous pressure are not routinely monitored, and peripheral vessels, such as the cephalic vein, rather than a central vein, are selected for drug infusion (16–18). Furthermore, endotoxin shock may not be the major cause of intra-operative hypotension. These findings underscore the needed for developing a simple system for closed-loop control of the infusion of NE suitable for use in veterinary clinics.

Anesthesia-associated hypotension is a major cause of intra-operative hypotension in companion dogs. Isoflurane is commonly used for the maintenance of general anesthesia in dogs undergoing surgery. The use of isoflurane can cause dose-dependent vasodilatory effects, leading to hypotension (19, 20). Isoflurane-induced hypotension in dogs has been successfully treated with NE in previous studies (16–18, 21).

Therefore, an automated closed-loop NE infusion system for the management of MAP in veterinary clinical settings was developed in this study. We hypothesized that our system allows stable NE infusion control and accurate control of MAP to a predefined target value when hypotension is induced during general anesthesia in dogs. This study aimed to assess the precision and stability of the system control for managing isoflurane-induced hypotension in Beagle dogs.

## 2 Materials and methods

### 2.1 Automated closed-loop NE infusion system

**Figure 1:**
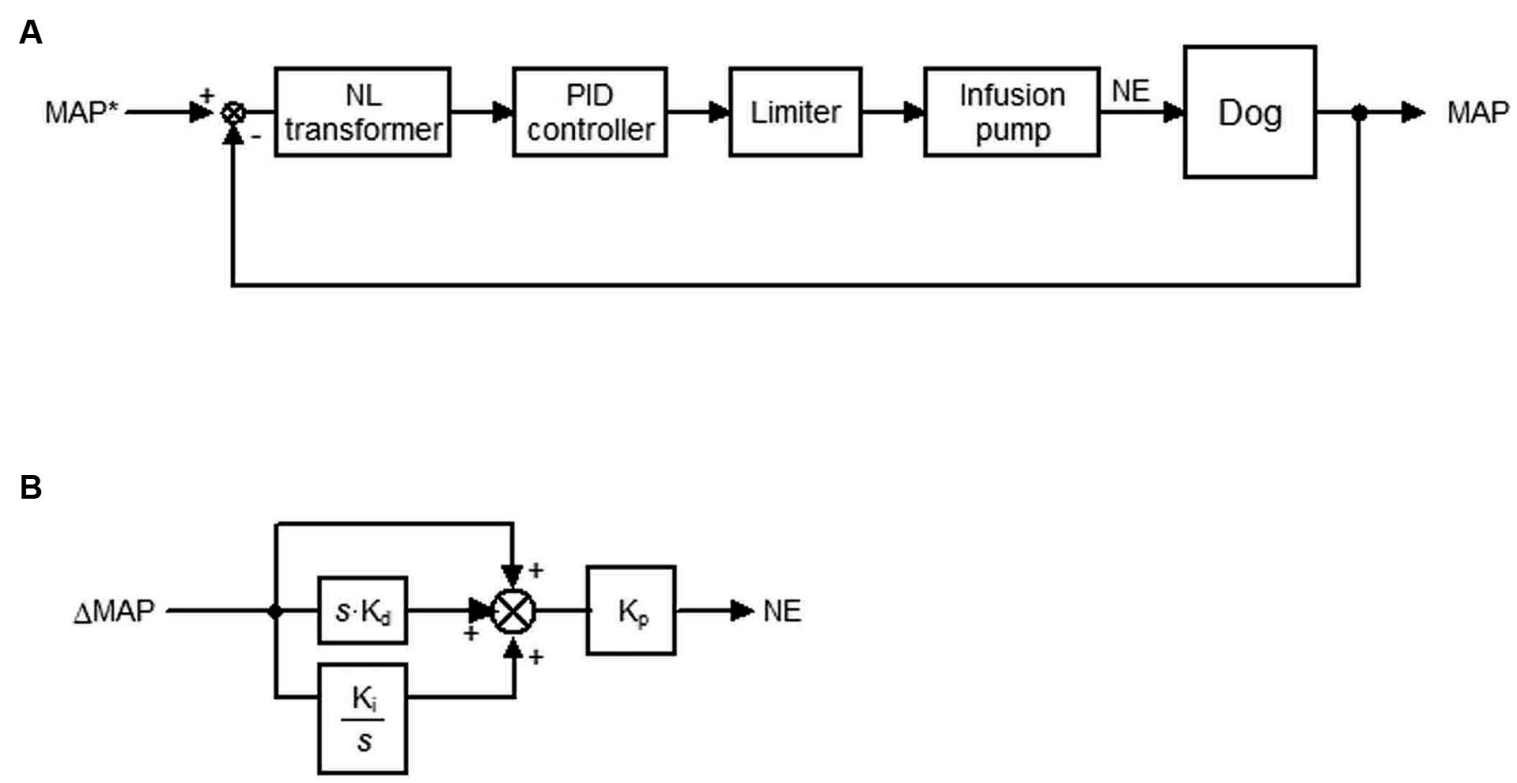
Closed-loop automated infusion system of norepinephrine (NE) to maintain the mean arterial pressure (MAP) in dogs with isoflurane-induced hypotension. **(A)** Block diagram of the system. tMAP represents the target MAP; NL, non-linear. A proportional-integral-derivative (PID) controller adjusts the infusion rate of NE to minimize the difference between MAP and tMAP (ΔMAP). **(B)** Block diagram of the PID controller. ΔMAP and the difference integrated with an integral gain (*K_i_*) and derivative gain (*K_d_*) are summed and scaled by the proportional gain (*K_p_*) to define the infusion rate of NE. *s* is a Laplace operator.

Figure 1 depicts a block diagram of the system developed in this study. The target MAP (tMAP) was defined by the user and input into the system (Figure 1A). The system titrated the infusion rate of NE such that the MAP was maintained at tMAP via a negative feedback mechanism.

The difference between tMAP and MAP (ΔMAP) in a non-linear (NL) transformer (Figure 1A) in cases wherein tMAP ≤ MAP is defined as


ΔMAP=tMAP−MAP

However, ΔMAP is amplified by an empirically-tuned sigmoid function of (tMAP – MAP) in cases wherein tMAP > MAP to correct hypotension more aggressively than hypertension. ΔMAP is defined as


$$\Delta \text{MAP}=\text{tMAP}–\text{MAP}+10–10/\left\{1+\exp\left[1.5\times \left(\text{tMAP}–\text{MAP}–5\right)\right]\right\}$$

The relationship between MAP and ΔMAP defined in the NL transformer is provided in Supplementary material S1.

A proportional-integral-derivative (PID) feedback controller was used to compute the infusion rate of NE to minimize ΔMAP defined in the NL transformer (Figure 1B). ΔMAP and the difference integrated with an integral gain (*K_i_*) and derivative gain (*K_d_*) were summed and scaled by a proportional gain (*K_p_*) in the PID feedback controller to compute the infusion rate. The gain constants for NE infusion [*K_p_* = 0.004 μg·kg^−1^·min^−1^·mmHg^−1^, *K_i_* = 0.006 s^−1^, *K_d_* = 96 s] were determined in a preliminary experiment based on the open-loop response of MAP to a step infusion of NE at 0.25 μg·kg^−1^·min^−1^ in four dogs (Supplementary material S2).

The infusion rate of NE computed using this method was processed in a limiter (Figure 1A) based on the following if–then rules:

If MAP > tMAP +10, then the infusion rate of NE is 0 μg·kg^−1^·min^−1^. Else if the computed infusion rate of NE is >1 μg·kg^−1^·min^−1^, then the infusion rate of NE is 1 μg·kg^−1^·min^−1^. Else, NE is administered at the computed infusion rate between 0 and 1 μg·kg^−1^·min^−1^.

The processed infusion rate of NE is sent to the infusion pump. The infusion rate of NE was updated every 2 s until the difference between tMAP and MAP disappeared.

### 2.2 Animals

The protocol for this study was approved by the Institutional Animal Care and Use Committee at the National Cerebral and Cardiovascular Center, Suita, Japan (ID: 22117 and 23013).

This study examined six consecutive female Beagle dogs aged 1.2 (1.1–1.3) years and weighing 9.1 (8.6–9.4) kg [median (interquartile range)]. All dogs were housed in a room on a 12:12 h light/dark cycle with controlled temperature (22 ± 1°C) and humidity (55 ± 15%). All dogs were considered healthy based on the medical history provided by the laboratory animal vendor and the findings of the physical examination performed by the investigators and institutional veterinary care team. The dogs were familiarized with handling and laboratory conditions by animal caretakers and investigators prior to commencing the study. The dogs were fed commercial dog food and fresh water was available *ad libitum*. The experiment was conducted after a 14-day acclimatization period. Food, but not water, was withheld for a minimum of 8 h prior to the induction of anesthesia.

### 2.3 Preparation

A 22-gauge (G) over-the-needle catheter (Insyte-W; Becton, Dickinson & Co., UT, United States) was placed in the cephalic vein. Anesthesia was induced with intravenous (IV) propofol (Propofol; Maruishi Pharmaceutical Co., Ltd., Osaka, Japan) administered to effect. All dogs were positioned in dorsal recumbency following endotracheal intubation. Anesthesia was maintained with 1.5% isoflurane delivered in an oxygen and air mixture at a flow rate of 2 L·min^−1^ with an inspired fraction of oxygen (FiO_2_) of 0.6 via a semi-closed rebreathing circle anesthetic system. The end-tidal carbon dioxide (PE’CO_2_) and oxygen saturation of hemoglobin (SpO_2_) were monitored using a multiparameter biological information monitoring device (Life Scope BSM-2391; Nihon Kohden Corp., Tokyo, Japan: Radical-7; Masimo Corporation, Irvine, CA, United States: MicroCapno50; Kimuramed, Tokyo, Japan) with a built-in automatic calibration system.

Mechanical ventilation was initiated immediately after the induction of anesthesia using a ventilator (PRO-45Va; ACOMA Medical Industry Co., Tokyo, Japan) in the A/C mode [setting: volume-controlled ventilation with tidal volume (VT), 10 mL·kg^−1^; respiratory rate (*f*R), 10–16 breaths·min^−1^; to maintain eucapnia of PE’CO_2_ 33–45 mmHg, 4.4–5.9 kPa]. The esophageal temperature was maintained at 37–38°C using a forced-air patient warmer (Circulating Thermal Water System, T-CARE; Kimuramed, Tokyo, Japan). Lactated Ringer’s solution (Lactec; Otsuka Pharmaceutical Factory, Inc., Japan) was infused at a dose of 5 mL·kg^−1^·h^−1^ via the infusion pump (Terfusion infusion pump TE-LM835A) throughout the study period.

**Figure 2:**
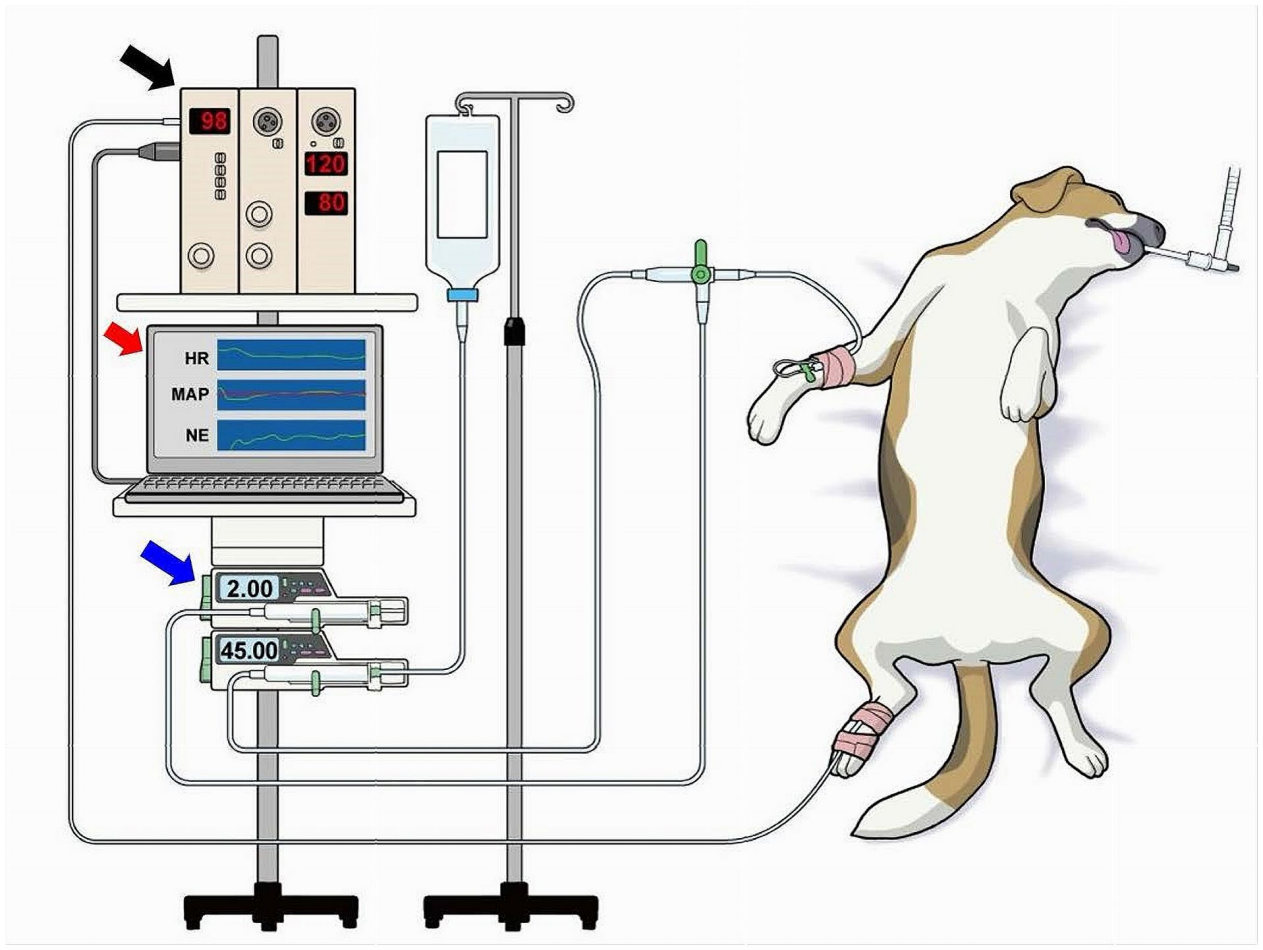
Overview of the experimental environment. A catheter was placed in the right dorsal pedal artery and connected to a biological amplifier (black arrow) via a noncompliant fluid-filled tubing and pressure transducer (not shown) to measure the mean arterial pressure (MAP). The amplifier was connected to a laboratory computer (red arrow) that was installed with the software of the closed-loop automated infusion system of norepinephrine (NE; Figure 1). NE was infused using a dedicated infusion pump (blue arrow) connected through a tube to the intravenous catheter via a three-way stopcock. The NE infusion rate was controlled by the laboratory computer.

A catheter (20 G, 30 mm length, BD Insyte-A; Becton, Dickinson & Co.) was placed in the right dorsal pedal artery and connected with fluid-filled noncompliant tubing to a pressure transducer (DX-200; Nihon Kohden, Tokyo, Japan) to measure AP (Figure 2). The transducer was placed at the level of the right atrium as a reference point for zero pressure (22, 23). Heparinized saline solution (4 IU·mL^−1^) was used to flush the arterial catheter, and infusion was commenced at a rate of 3 mL·h^−1^ with 300 mm Hg pressure applied to the infuser bag (22, 23). Surface ECG was recorded to monitor the heart rate (HR). A biological amplifier (AB-601G, AP-641G, Nihon Kohden, Tokyo, Japan) was used to amplify the analog signals of ECG, HR, and AP.

The analog signals of ECG, HR, and AP were digitized using a laboratory computer (LC-72 N10, Logitec, Tokyo, Japan) at 200 Hz with a 16-bit analog-to-digital converter [AD16-16 U(PCI)EV, Contec, Japan] throughout the experiment. The digitized signals were stored on a hard disk for offline analysis. The digitized signal of AP was smoothed by a low-pass filter (time constant, 10 s) in the closed-loop control and used as the system-controlled MAP. An externally controllable syringe pump (Terufusion Syringe Pump TE-SS835N; Terumo Corporation, Tokyo, Japan) loaded with a 20-mL syringe filled with saline and 0.5 mg·mL^−1^ of NE that was connected through extension tubing to the intravenous catheter by a three-way stopcock was used to infuse NE (Figure 2). The infusion pump was controlled by the laboratory computer via the RS232C interface (Figure 2).

### 2.4 Study protocol

**Figure 3:**
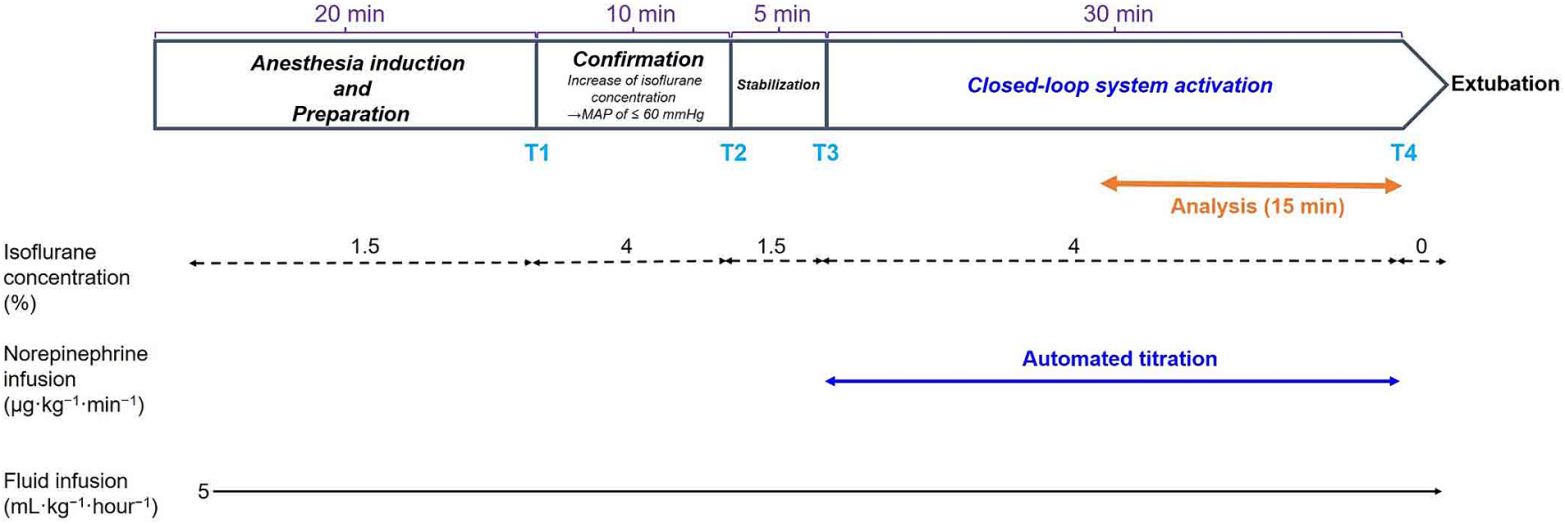
Time course of the experimental protocols. Values of isoflurane concentrations are the median values of six dogs. T1: 0 min in initial hypotension trial; T2: 10 min in initial hypotension trial; T3: 0 min in hypotension with system activation; T4: 30 min in hypotension with system activation.

Figure 3 presents the study protocol. After general anesthesia was induced, but before our system collected the data on the closed-loop MAP control, the concentration of isoflurane was increased to 4% (T1 in Figure 3) and maintained for 10 min until isoflurane-induced hypotension (MAP ≤60 mmHg) was reached. The concentration of isoflurane was slightly adjusted according to an individual’s MAP response. Then, the isoflurane concentration was decreased to 1.5% (T2 in Figure 3) when the MAP was observed to decrease to ≤60 mmHg. The concentration of isoflurane was maintained at 1.5% for 5 min (from T2 to T3 in Figure 3) to enable stabilization; the baseline data for MAP were acquired at the end of stabilization. The concentration of isoflurane was increased to 4% (T3 in Figure 3) to induce hypotension in the dogs, and the system was activated by closing the feedback loop of NE infusion. The tMAP value was set to 60 mmHg during system activation based on the current definition of hypotension in dogs (24, 25). The performance of the closed-loop control by the system was observed for 30 min (from T3 to T4 in Figure 3) after system activation.

In addition, the system’s performance was observed for 60 min in one of six dogs to evaluate the precision and stability of the closed-loop control for a longer period (not indicated in Figure 3).

### 2.5 Post-experimental veterinary care

IV cefazolin sodium (22 mg·kg^−1^; cefazolin sodium; Nichi-Iko Pharmaceutical Co., Ltd., Toyama, Japan) was administered before extubation to minimize possible infection and discomfort due to skin and vascular puncture. In addition, anti-inflammatory topical ointment (DEXAN-VG OINTMENT 0.12%; Fuji Pharma Co., Ltd., Toyama, Japan) was applied at the catheterization site. All dogs were returned to their institutional kennel after complete recovery. The post-experimental daily observation was performed by the investigators and the institutional veterinary care team.

### 2.6 Off-line data analysis

HR and MAP were determined every 10 s during the 30-min period of system activation.

#### 2.6.1 Performance of the closed-loop NE infusion system

The precision and stability of the control by the system were analyzed over the second half of the 30-min period, i.e., from 15 to 30 min after system activation since the first half of the 30-min period included the MAP reduction from its baseline normal value followed by dynamic changes in MAP and the NE infusion rate until they stabilized. Thus, data from 90 time points were analyzed for each dog. An acceptable range of MAP was defined as target MAP ±5 mmHg, i.e., 55 ≤ MAP ≤65, according to a previous study (26). The percentage of time during which MAP was maintained within the acceptable range was calculated as follows: numbers of the time points with MAP in the acceptable target range divided by the total number of observations (=90). Percentages of >85% were considered acceptable.

In addition, the precision and stability of the closed-loop system were assessed using previously described parameters (27, 28). The percentage performance error (PE), median performance error (MDPE), median absolute performance error (MDAPE), wobble, and divergence in the control of MAP in each dog were calculated as follows.

#### 2.6.2 Performance error

PE is defined as a measure of the difference between each measured value of MAP and the target value. It is presented as a percentage of the target value as follows:


$$PEi=\frac{\left(\text{measured}\;{\text{MAP}}_{i}-60\right)}{60}\times 100$$

where PE*_i_* is the percentage performance error at the *i*th data point and measured MAP*_i_* is the measured MAP at the *i*th data point.

#### 2.6.3 MDPE

MDPE is the median of all PE values for each dog. It indicates whether the measured values for MAP are below or above the target MAP value.


$$\text{MDPE}=\text{median}\;\left\{PEi,\;i=1,\ldots ,90\right\}$$

#### 2.6.4 MDAPE

MDAPE is a measure of the median of the absolute values of PE (|PE|). It represents an average of the magnitudes of the differences if the measured values for MAP are below or above the target value.


$$\text{MDAPE}=\text{median}\;\left\{|PEi|,\;i=1,\ldots ,90\right\}$$

#### 2.6.5 Wobble

Wobble is a measure of the intraindividual variability of PE around MDPE.


$$\text{WOBBLE}=\text{median}\;\left\{|PEi-\text{MDPE}|,\;i=1,\ldots ,90\right\}$$

#### 2.6.6 Divergence

Divergence shows the trend of changes in |PE| over the investigation time. It indicates whether the differences in magnitude between the target and measured value for MAP decrease or increase over the investigation time.


$$\text{DIVERGENCE}=\frac{90\times \sum_{i=1}^{90}\left(Ti\times \left|PEi\right|\right)-\sum_{i=1}^{90}Ti\times \sum_{i=1}^{90}\left|PEi\right|}{90\times \sum_{i=1}^{90}Ti^{2}-{\left(\sum_{i=1}^{90}Ti\right)}^{2}}\text{.}$$

where *i* = *i*th single measurement of the investigation period, and *T* is time in min.

### 2.7 Statistics

Because this is a feasibility study, we did not estimate a formal sample size. Instead, the sample size was determined based on a recent study of the closed-loop drug infusion system (29). Continuous data are presented as median (interquartile range) or number (%) unless otherwise indicated. All statistical analyses were performed using Microsoft Office Excel 2016.

## 3 Results

Table 1 presents the baseline characteristics of the six dogs.

**Table 1:** Baseline characteristics of the six dogs.

Age (y)	1.2 (1.0–1.3)
Body weight (kg)	9.1 (8.6–9.4)
Baseline MAP (mmHg)	92 (89–95)
Baseline heart rate (beats·min^−1^)	123 (115–137)

Values are median (interquartile range). MAP, mean arterial pressure.

### 3.1 Automated MAP control by computer-controlled closed-loop NE infusion system

**Figure 4:**
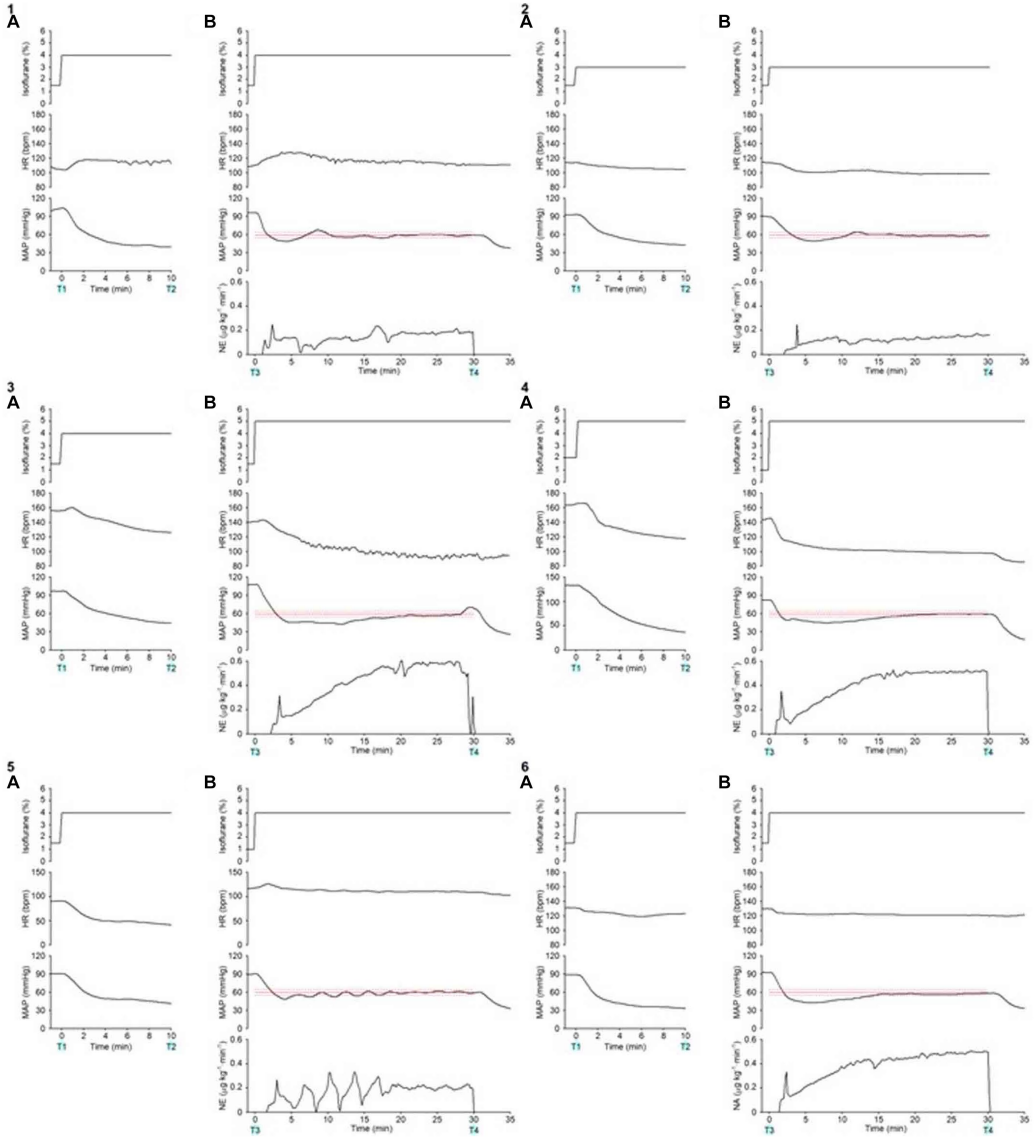
Experimental time courses in a representative dog (4–1) and 5 other dogs (4–2, 4–3, 4–4, 4–5, 4–6) validate the ability of the closed-loop automated norepinephrine (NE) infusion system for maintaining mean arterial pressure (MAP). **(A)** Time course of the inhaled isoflurane concentration, heart rate (HR), and mean arterial pressure (MAP) when the concentration of isoflurane was increased at 0 min without the system activation. **(B)** Time course of isoflurane concentration, HR, MAP, and infusion rate of norepinephrine (NE) when the concentration of isoflurane was increased and the system was activated at 0 min. The red line represents the target MAP value (60 mmHg). The red broken lines represent the acceptable target range of MAP (55–65 mmHg). T1: 0 min in initial hypotension trial; T2: 10 min in initial hypotension trial; T3: 0 min in hypotension with system activation; T4: 30 min in hypotension with system activation.

Figures 4-1 presents the time course of the experimental data of a representative dog (dog no. 2 in Tables 2, 3). MAP decreased gradually and was lowered to 40 mmHg 10 min after increasing the concentration of isoflurane from 1.5 to 4% at 0 min without activating the system (from T1 to T2 in Figures 4-1A). In contrast, MAP was temporarily decreased to 49 mmHg when the concentration of isoflurane was increased from 1.5 to 4%, and the system was activated simultaneously at 0 min (T3 in Figures 4-1B). However, MAP gradually recovered to the target value of 60 mmHg following an automated increase in the infusion rate of NE. MAP was maintained within the predefined acceptable target range accurately and stably during the second half of the 30 min period, i.e., from 15 to 30 min after system activation (Figures 4-1B). MAP decreased, returning to 40 mmHg, once the system was deactivated at 30 min (T4 in Figures 4-1B), indicating the system’s efficacy in preventing hypotension.

**Table 2:** MAP from 15 to 30 min after system activation.

		Percentage of time (%)		
Dog number	MAP (mmHg)	55 ≤ MAP ≤65	MAP >65 mmHg	MAP <55 mmHg
1	58 ± 1	100	0	0
2	58 ± 2	91	0	9
3	57 ± 5	63	9	28
4	58 ± 2	89	0	11
5	60 ± 2	100	0	0
6	57 ± 1	100	0	0
Overall	58 (57–58)	96 (89–100)	0 (0–0)	4 (0–11)

MAP, mean arterial pressure. Acceptable target range of MAP: 55 ≤ MAP ≤ 65 mmHg. MAP in each dog was the time averaged from 15 to 30 min after system activation, and represented as mean ± SD. The overall group data are presented as median (interquartile range).

**Table 3:** Performance index values for closed-loop NE infusion system and NE infusion rate during 15 to 30 min after system activation.

Dog number	MDPE (%)	MDAPE (%)	Wobble (%)	Divergence (%·min^−1^)	NE dose (μg·kg^−1^·min^−1^)
1	−3.6	3.6	0.5	0.20	0.14 ± 0.02
2	−2.2	2.2	1.6	−0.34	0.18 ± 0.03
3	−6.1	6.5	2.0	−0.33	0.53 ± 0.11
4	−1.8	1.8	1.1	−0.62	0.50 ± 0.05
5	−0.2	1.9	1.9	−0.15	0.20 ± 0.03
6	−5.1	5.1	0.7	−0.10	0.47 ± 0.04
Overall	−2.9 (−4.7 to −1.9)	2.9 (2.0–4.7)	1.3 (0.8–1.8)	−0.24 (−0.34 to −0.11)	0.33 (0.18–0.49)

PE, percentage performance error; MDPE, median performance error; MDAPE, median absolute performance error; NE: norepinephrine. Overall group data are presented as median (interquartile range). NE in each dog was the time averaged from 10 to 30 min after system activation, and represented as mean ± SD.

**Figure 5:**
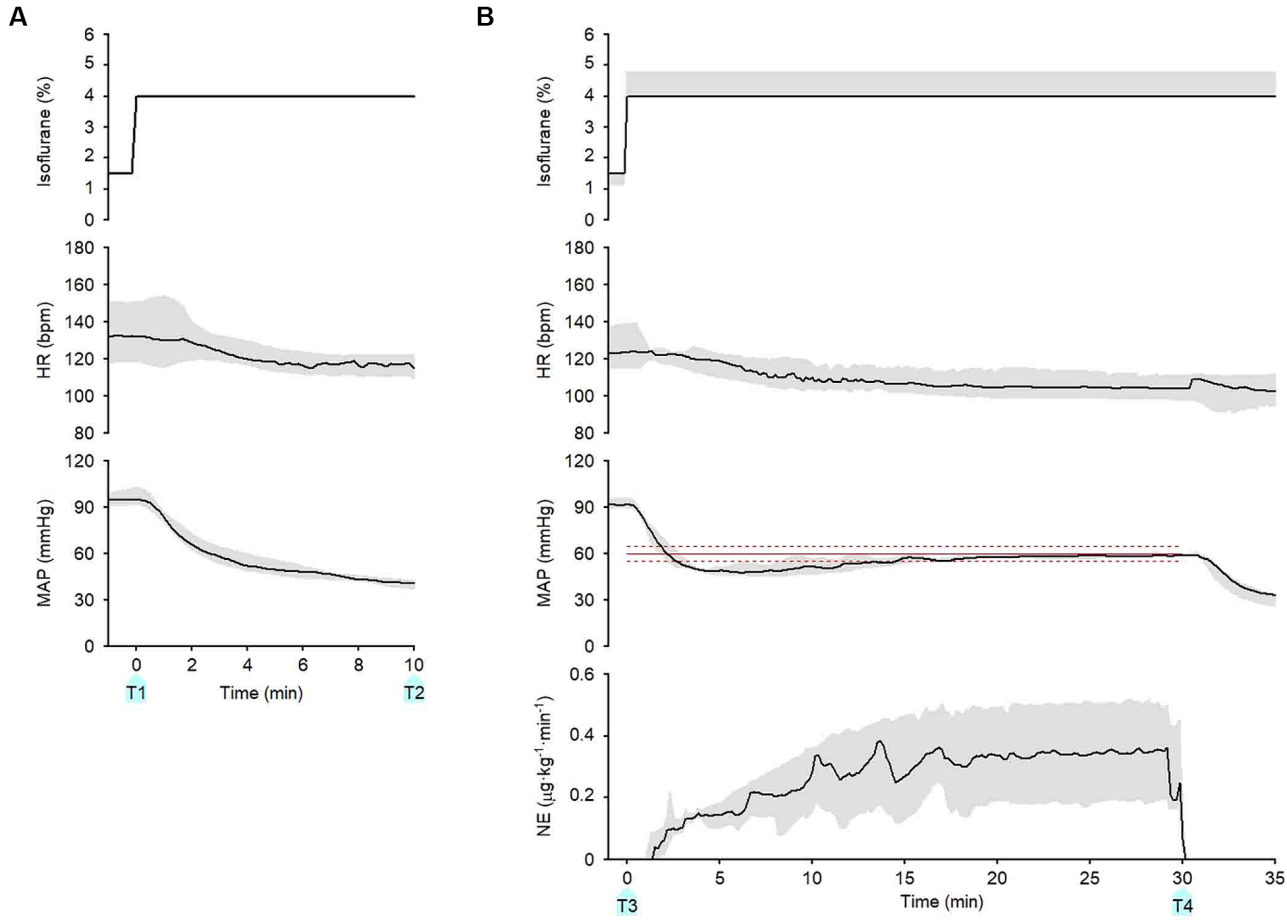
Summary of experimental time courses of the six dogs to validate the ability of the closed-loop automated infusion system for administration of norepinephrine (NE) to maintain mean arterial pressure (MAP). **(A)** Time course of isoflurane concentration, heart rate (HR), and MAP when the concentration of isoflurane was increased at 0 min without activating the system. **(B)** Time course of isoflurane concentration, HR, MAP, and infusion rate of NE when the concentration of isoflurane was increased, and the system was activated at 0 min. The black solid line and the gray shade represent the median and interquartile range, respectively. The red line represents the target MAP value (60 mmHg). The red broken lines represent an acceptable target range of MAP (55–65 mmHg). T1: 0 min in initial hypotension trial; T2: 10 min in initial hypotension trial; T3: 0 min in hypotension with system activation; T4: 30 min in hypotension with system activation.

**Figure 6:**
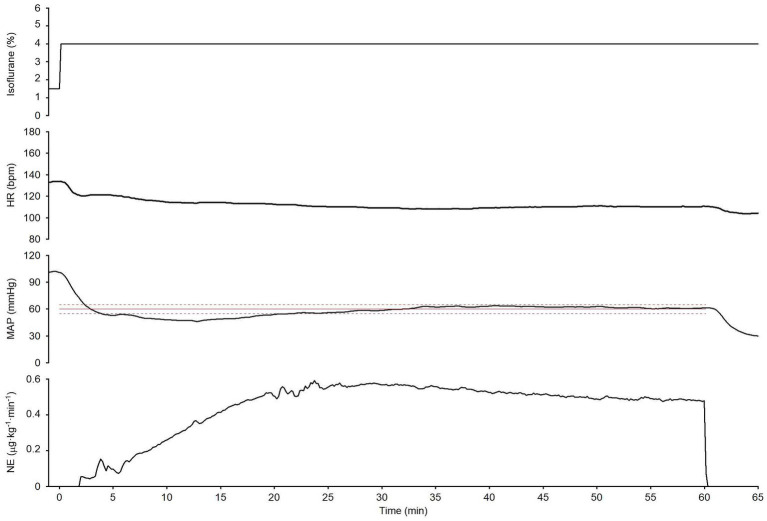
An experimental time course in one dog evaluates the ability of the closed-loop automated norepinephrine (NE) infusion system to maintain mean arterial pressure (MAP) over a long period (60 min). The time course shows the isoflurane concentration, HR, MAP, and NE infusion rate when the isoflurane concentration was increased; the system was activated at 0 min. The red line is the target MAP value (60 mmHg). The broken red lines indicate the acceptable MAP target range (55–65 mmHg).

Figures 4-2 presents the time course of a dog’s experimental data (dog no. 1 in Tables 2, 3). As with the representative dog, MAP control was stable and accurate. After system deactivation at 30 min (T4), trace data was not recorded in this animal because of an unexpected freeze of the analog-to-digital conversion in the laboratory computer; however, we noted an abrupt decrease in MAP. Figures 4-3 presents the time course for dog no. 3 in Tables 2, 3. The recovery of MAP to the acceptable range (55 ≤ MAP ≤65 mmHg) was relatively slow in this animal, but MAP was controlled from 20 to 30 min after system activation. Figures 4-4 presents the time course of dog no. 4 in Tables 2, 3. MAP control was stable and accurate in this animal, similar to the representative dog. Figures 4-5 presents the time course of dog no. 5 in Tables 2, 3. In this animal, a minor oscillation in MAP and the NE infusion rate was noted during the first half of the observation period and gradually subsided; MAP was controlled to the acceptable range during the second half. Figures 4-6 presents the time course of dog no. 6 in Tables 2, 3. This animal’s MAP control was stable, similar to the representative dog.

Figure 5 summarizes the time course of the experimental data obtained from the six dogs. After the **isoflurane concentration was increased** from 1.5 (1.5–1.5)% to 4 (4–4)% at 0 min (T1) without the system activated, MAP decreased from 95 (91–99) to 41 (37–42) mmHg at 10 min (T2; Figure 5A). In contrast, after the concentration of isoflurane was increased from 1.5 (1.0–1.5)% to 4 (4–4.8)% with the system activated at 0 min (T3), MAP temporarily decreased to 47 (43–49) mmHg (Figure 5B). However, MAP gradually recovered to the target MAP following an automated increase in the infusion rate of NE in all six dogs. The median MAP was maintained within the predefined acceptable target range during the second half of the 30-min period (Figure 5B). ECG monitoring did not detect any arrhythmia during the closed-loop control of NE infusion in any dogs.

### 3.2 Performance of the computer-controlled closed-loop NE infusion system

Table 2 summarizes the percentage of the time during the second half of the 30-min period when MAP was maintained within the acceptable target range of MAP (55–65 mmHg) in the six dogs. MAP was maintained in the acceptable range for >85% of the 15-min period in five of the six dogs. Table 3 summarizes the performance parameters of the control of MAP by this system in the six dogs. MDAPE and wobble were < 5%, indicating the accuracy and stability of the control of MAP by this system. The median and interquartile range of divergence was negative in the six dogs, indicating that the error in the MAP control reduced with time. The median infusion rate of NE in the six dogs from 15 to 30 min after system activation averaged 0.33 (0.18–0.49) μg·kg^−1^·min^−1^.

### 3.3 Performance of the system over a longer duration

The system’s performance was observed for 60 min in one dog (dog no. 6) to evaluate the stability of the closed-loop control over a long period (Figure 6). MAP temporarily decreased to less than the target MAP (60 mmHg) when the isoflurane concentration was increased from 1.5 to 4%, and the system was simultaneously activated at 0 min; however, it gradually recovered to the target MAP following an automated increase in the NE infusion rate. MAP was maintained within the predefined acceptable target range 85% of the time, from 15 to 60 min after system activation (Figure 6). In this observation, MDPE, MDAPE, Wobble, and Divergence were 1.8%, 4.1%, 2.9%, and − 0.23%·min^−1^, respectively. These results indicate that MAP was maintained at the target MAP (60 mmHg) with acceptable accuracy and stability for a long period. Negative divergence indicated that the error in MAP control was reduced with time during the observation period (i.e., 15–60 min after system activation).

### 3.4 Recovery from anesthesia

Recovery from isoflurane anesthesia and extubation was uneventful in all six dogs. All dogs were returned to their kennel in the institution after complete recovery. No complications were observed in any of the dogs.

## 4 Discussion

### 4.1 Interpretation

To our knowledge, this feasibility study in a likely setting of veterinary clinics is the first to investigate the precision and stability of a computer-controlled closed-loop NE infusion system in maintaining MAP within a predetermined acceptable target range in Beagle dogs with isoflurane-induced hypotension. The study findings suggest that this system may efficaciously prevent intra-operative hypotension induced by isoflurane inhalant anesthesia in dogs. Furthermore, this system may reduce the stress and work imposed on the veterinary practitioner, especially at private clinics where veterinary anesthesiologists may be unavailable. These results strongly warrant further development of this system for its reliable translation to daily veterinary practice.

Several closed-loop NE infusion systems have been developed to aid in managing AP in preclinical settings under various hemodynamic conditions (14, 15, 30–32). Some of these systems have already been used in humans (13, 26, 33). The system developed in this study is a simplified version of the infusion system that we developed previously (14, 15). Our previous system required central venous catheterization and monitoring of multiple hemodynamic variables, which may not be feasible in current common clinical practice for companion dogs. In contrast, the present system only requires invasively measured MAP for the instrumentation (Figure 2). The findings of the present study indicate that titration of NE infusion rate by the system developed in this study led to the maintenance of MAP within ±5 mmHg of the predetermined target value for >85% of the observation period in five of six Beagle dogs with isoflurane-induced hypotension. The present study’s findings could not be compared with those of relevant studies in veterinary medicine because no studies are available.

We chose PID control because it is the most popular method for regulating actuators in the negative feedback loops in common automated systems and the closed-loop control of cardiovascular drug infusion in managing hemodynamically unstable dogs (34). Tuning procedures of the PID controller gain constants are well established and effective, as in Chien’s method adopted in our study (see Supplementary material S2). This system’s NE infusion rate has lower and upper bounds (0 and 1 μg·kg^−1^·min^−1^, respectively). The NE infusion is stopped when the MAP is greater than the target MAP +10 mmHg (i.e., 70 mmHg). However, rather than stopping the NE infusion at the high MAP, implementing an anti-windup loop would enable stable and rapid control of MAP. For example, error integration can be clamped once the NE rate computed by the PID controller is beyond the lower or upper bound. The control loop of our system needs further development and refinement before it can be applied to daily veterinary clinical practice.

In a previous experimental study using a porcine model of vasodilator-induced hypotension, MAP was maintained within the target range via manual titration of NE infusion for only 14% of the time (26). However, the percentage of time during which closed-loop control of NE infusion optimized MAP to within the target range was reported to be >90% in previous clinical and experimental studies (26, 35). The percentage of time during which our system successfully maintained MAP within the target range in this study seemed to be higher than that achieved by manual titration in a previous study (26). Moreover, this percentage was comparable with that noted in previous closed-loop control systems of NE infusion in human and porcine (11, 26, 33).

Notably, the observation period of up to 30 min after system activation used in the present study was short compared with those used in previous studies (45–120 min of observation period) (26, 35). However, the 30-min observation period may be sufficient to assess the performance of this system. The median divergence, the trend of the absolute difference between the target and measured value for MAP, was negative in the present study (Table 3). Thus, the error in MAP control was progressively reduced even during the short observation period. The performance of this system was observed for a longer period (60 min) in an additional experiment (conducted in dog number 6 in Figure 6), and it was found that the precision and stability of MAP control by this system were acceptable. MAP was maintained within the acceptable range (target MAP ±5 mmHg) for 85% of the time from 15 to 60 min after system activation. Moreover, the control performance parameters, including MDPE, MDAPE, Wobble, and Divergence, were comparable with those reported in previous studies (26, 35).

Varvel’s performance criteria are commonly applied to the entire run of the closed-loop system (26, 27); however, the criteria were applied only to the last 15 min of the closed-loop control in this study. We omitted the first 15 min because they included the initial MAP reduction from its baseline value to the target MAP and bottom MAP (see Figure 5). Varvel’s performance criteria would be erroneously rated if we included this period when the animal’s baseline MAP was high.

From 15 to 30 min after system activation, the median (IQR) percentage of the time with MAP <60 mmHg was 89 (88–98)% in the 6 dogs. The median (IQR) percentage of the time with 55 ≤ MAP <60 mmHg was 85 (66–89)% in the 6 dogs. This finding indicates that MAP was controlled close to the target MAP (60 mmHg) but with residual positive control error (target MAP > measured MAP). However, the hypotensive state can be eradicated by defining the target MAP values >60 mmHg with a safety margin.

The primary mechanism underlying isoflurane-induced hypotension is the suppression of the activity of the sympathetic nervous system (deactivation of the α- and β-adrenergic receptors) and the consequent reduction of systemic vascular resistance (SVR) (36, 37). MAP was lowered to <50 mmHg in all dogs in the present study in response to the increase in the concentration of isoflurane without system activation (Figure 5A). Anesthesia-induced hypotension can be treated with fluid challenges in dogs (25). However, in isoflurane-induced hypotension, activation of the α- and β-adrenergic receptors with use of NE may be a mechanistically more reasonable approach to effectively maintain SVR and AP (38, 39).

Thus, NE infusion probably did not negatively affect organ perfusion or cardiac function during the study period in the present study. However, the organ perfusion status during NE infusion was not evaluated using dynamic indices and specific surrogate markers, such as blood lactate levels, as the period of NE titration was very short, i.e., ≤ 30 min, and the research purpose was to confirm the precision and stability of the system for maintaining MAP within the predetermined acceptable target range. As described earlier, an automated closed-loop vasopressor infusion system is better at maintaining a stable MAP without overshooting the NE infusion rate.

This closed-loop automated NE infusion system for optimizing MAP may be further developed into an autonomous closed-loop intervention system that automatically optimizes the total hemodynamic conditions of human and veterinary patients.^1^ Simple but fully autonomous hemodynamic management could be used during perioperative periods, hospital wards, and in intensive care settings.

### 4.2 Generalizability and limitations

This feasibility study was conducted to test our hypothesis in healthy laboratory Beagle dogs under specific experimental conditions. Thus, the results obtained at this stage may not be sufficiently generalizable enough to conclude that this system offers universally reliable control of AP during surgical anesthesia in companion dogs with some diseases.

This study has potential limitations that should be taken into consideration. First, formal sample size estimation was not conducted as this was a feasibility study. Although the present sample size enabled us to test our concept and confirm the precision and stability of the system, further studies with an adequate sample size must be conducted to validate these results.

Secondly, although previous studies in humans and porcine have demonstrated that a computer-controlled closed-loop vasopressor infusion system has advantages over manual titration (13, 40), the precision and stability of the MAP control by our system could not be compared with those relating to the manually-titrated infusion of NE. To investigate this issue, definitive studies must be conducted in the future, preferably with a longer observation period.

Thirdly, the PID parameters in this system remain fixed, assuming that the parameters based on the population-averaged NE responses (Supplementary material S2) are universally applicable to different dogs. However, drug responses can vary both between and within dogs. This can cause imprecision and instability in controlling MAP through the system. Indeed, the MDAPE values of two of the six dogs were a little above 5%, and minor oscillation in MAP was observed in another dog. An adaptive control schema may effectively address these issues and achieve further accuracy in controlling MAP and reducing the PE values. One of the candidate schemas for adaptive control is an adaptive PID controller utilizing a Fuzzy logic algorithm (41, 42). In this schema, the default PID gain constants are derived from the population-averaged response of the MAP to NE, as in the present study. The gain constants are periodically updated during the system activation by evaluating individual responses of the MAP to NE, e.g., the settling time and the overshoot of the MAP. The periodic updates would use Fuzzy logic if–then rules based on the experts’ experiences and knowledge of MAP control by NE. This schema can effectively couple the advantages of control engineering and the human experience.

Finally, the precision and stability of the system under various disease conditions under different hemodynamic states was not investigated. Furthermore, isoflurane is not ordinarily used alone; analgesics, such as opioids, are co-administered during surgical anesthesia in companion dogs. Thus, it is unclear whether our system works correctly when multiple drugs are administered simultaneously. Further studies must be conducted to examine whether such other factors may affect the operation of the system.

## 5 Conclusion

The system developed in the present study was able to titrate the NE infusion rate to maintain MAP within the predetermined target range in dogs with isoflurane-induced hypotension in an accurate and stable manner. Further evaluations, including comparison of the performance of the system with that of manual control of NE infusion over a longer period must be conducted to assure a reliable translation of the use of this system in the clinical care of companion dogs.

## Data availability statement

The original contributions presented in the study are included in the article/Supplementary material, further inquiries can be directed to the corresponding authors.

## Ethics statement

The animal study was approved by the Institutional Animal Care and Use Committee at the National Cerebral and Cardiovascular Center, Suita, Japan (ID: 22117and 23013). The study was conducted in accordance with the local legislation and institutional requirements.

## Author contributions

KSs: Conceptualization, Data curation, Formal analysis, Funding acquisition, Investigation, Methodology, Writing – original draft, Writing – review & editing. TK: Methodology, Software, Writing – review & editing. HMa: Investigation, Writing – review & editing. SY: Investigation, Writing – review & editing. MK: Investigation, Writing – review & editing. AY: Investigation, Writing – review & editing. YY: Investigation, Writing – review & editing. HMo: Investigation, Writing – review & editing. KSt: Investigation, Writing – review & editing. TN: Methodology, Software, Writing – review & editing. AK: Writing – review & editing. YK: Methodology, Writing – review & editing. JA: Methodology, Writing – review & editing. KSk: Funding acquisition, Investigation, Writing – review & editing. TI: Funding acquisition, Writing – review & editing. KU: Conceptualization, Data curation, Formal analysis, Funding acquisition, Investigation, Methodology, Project administration, Resources, Software, Supervision, Validation, Visualization, Writing – original draft, Writing – review & editing.

## Footnotes

^1^ https://vimeo.com/897450724/a410ec56fb
