# Supplementary material for: Computer-controlled closed-loop norepinephrine infusion system for automated control of mean arterial pressure in dogs under isoflurane-induced hypotension: a feasibility study
Source: Front Vet Sci. 2024 May 31;11:1374356. doi: 10.3389/fvets.2024.1374356 (PMC11177754; doi:10.3389/fvets.2024.1374356)
Supplement: Supplementary file 1 [file Data_Sheet_1.PDF]

Supplementary Material

S1

Mean arterial pressure (MAP) and difference between target MAP (tMAP) and MAP (ΔMAP) defined in a non-linear (NL) transformer in the system

Solid line in supplementary Figure 1 presents the relation between MAP and Δ MAP defined in the NL transformer in the system (Figure 1a in main text). Dotted line in the supplementary Figure 1 presents the relation between MAP and tMAP − MAP (tMAP = 60 mmHg). To correct hypotension more aggressively than hypertension, the NL transformer amplifies Δ MAP in cases wherein MAP < tMAP (arrows).

Supplementary Figure 1

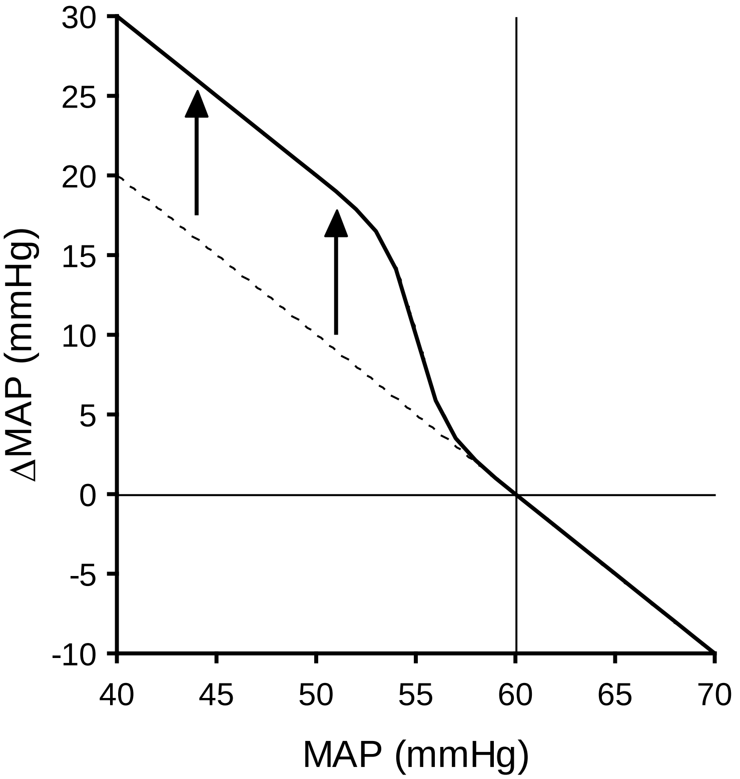

S2

Response of MAP to infusion of norepinephrine (NE)

The response of MAP to the infusion of NE was examined in four dogs aged 1.2 (1.2–1.2) years weighing 10.5 (10.2–10.6) kg under open-loop conditions. NE was infused in a stepwise manner at a rate of 0.25 μg·kg^{-1}·min^{-1} for 10 min to obtain a step response of MAP.

Data Analysis

Evaluation of the response of MAP and the design of the proportional-integral-derivative **(***PID***)**

controller

The step response of MAP was described using a transfer function of a first-order model with a transport delay. The change in MAP from baseline (δMAP) in response to NE infusion in this model can be expressed using the following formula:

$$\delta MAP = G \cdot [1\text{-}Exp(\frac{t\text{-}L}{T})] \quad t > L \qquad \text{(Supl.1)}$$

$$\delta MAP = 0 \qquad t \leq L \qquad \text{(Supl.2)}$$

where G is static gain [mm Hg·(μg·kg^{-1}·min^{-1})$^{-1}$], L is the transport delay (s), and T is time constant (s). The parameters of the transfer function of MAP response were averaged for the four dogs. The averaged parameters were used to determine the PID gain constants, K_p, K_i, and K_d, in accordance with the method described by Chien et al. (1). Their method is usually called the “CHR method.” Compared with other methods such as the Ziegler-Nichols method for PID gain tuning, the CHR method often results in more conservative tuning, leading to more stable but less aggressive control performance.

Results

Supplementary Figure 2 presents the open-loop responses of MAP to the infusion of NE, that is, the median time course of δMAP during NE infusion (n = 4). The fit of the measured δMAP to *Eq. Sup1.1* indicates that the averaged values of G, L, and T are 125 ± 35 mmHg·(μg·kg^{-1}·min^{-1})$^{-1}$, 192 ± 61 s, 154 ± 91 s. The PID gain constants for NE infusion [K_p = 0.004 μg·kg^{-1}·min^{-1}·mmHg^{-1}, K_i = 0.006 s^{-1}, K_d = 96 s] were determined based on these averaged parameters.

Supplementary Figure 2

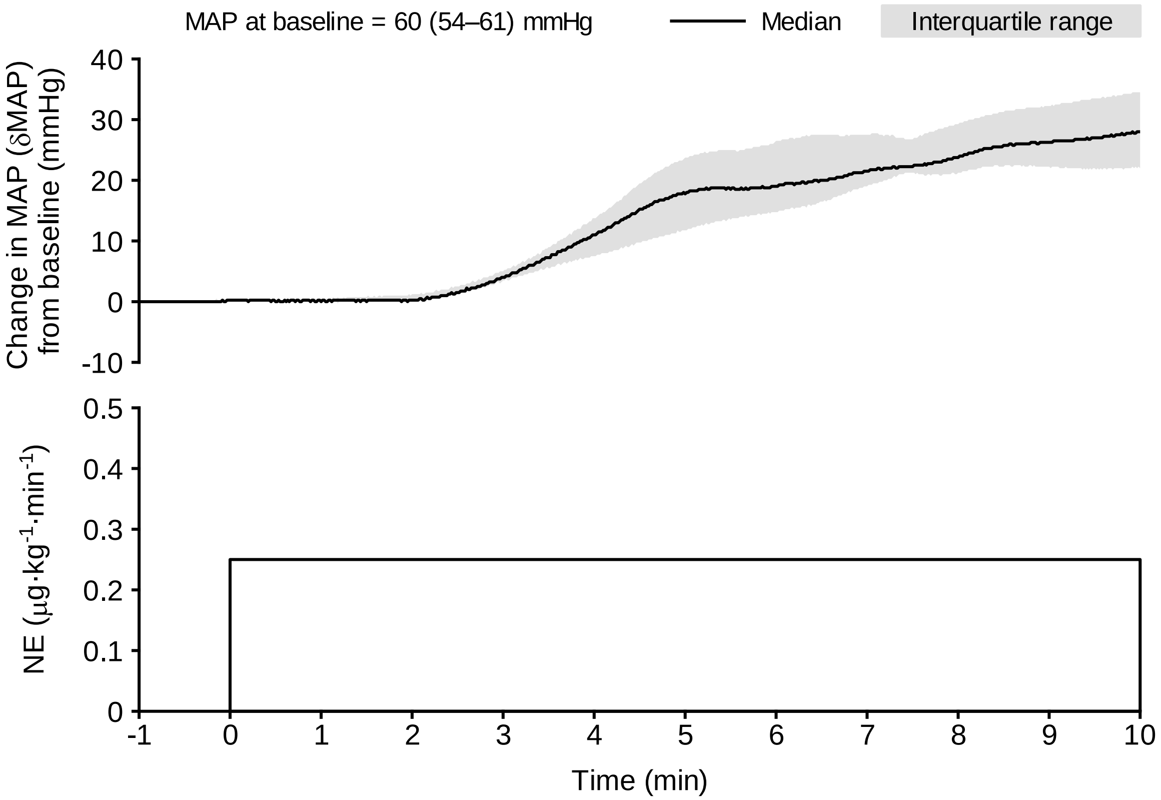

References

1. Chien KL, Hrones JA, Reswick JB. On the automatic control of generalized passive systems. Trans ASME. (1952) 74:175-85.
